# Implementation of a Standardized Comprehensive Assessment Tool in France: A Case Using the InterRAI Instruments

**DOI:** 10.5334/ijic.3297

**Published:** 2018-04-18

**Authors:** Matthieu de Stampa, Valérie Cerase, Emmanuel Bagaragaza, Elodie Lys, Quentin Alitta, Cedric Gammelin, Jean-Claude Henrard

**Affiliations:** 1 Assistance Publique Hôpitaux de Paris, Hospitalisation à Domicile, Unité Mixte de Recherche (UMR) 1168 INSERM, UVSQ, VIMA (Vieillissement et Maladies Chroniques), InterRAI France, Paris, FR; 2 Institut Maladie Alzheimer (IMA), Centre Départemental de Gérontologie, interRAI France, Marseille, FR; 3 Pôle Recherche SPES « Soins Palliatifs En Société », Maison Médicale Jeanne Garnier, Unité Mixte de Recherche (UMR) 1168 INSERM, UVSQ, VIMA (Vieillissement et Maladies Chroniques), InterRAI France, Paris, FR; 4 Centre Départemental de Gérontologie, InterRAI France, Marseille, FR; 5 Université de Versailles, Saint-Quentin en Yvelines, InterRAI France, Paris, FR

**Keywords:** standardized comprehensive assessment, integrated model of care, interRAI instruments, long term care

## Abstract

**Background:**

The improvement of quality of care requires a standardized and comprehensive assessment tool but implementation is challenging.

**Purpose:**

We have reported on the development of the interRAI instruments in France from the onset to the mandatory use at the national level. We also have identified in the literature and in practices, incentives and barriers for the implementation of this integrated clinical information system in long term care.

**Results:**

Three periods in the interRAI instruments development were identified over the last twenty years. The first one was a research approach about improving quality of long term care. The second one was an experimental clinical use into an integrated care model with case management. The third one was a call for tenders issued by a French national agency, and the choice to use the interRAI-HC (Home Care) for all case managers. The main incentives and barriers that were identified include the national context, the target population, the providers involved and the impact on their practice, the interRAI instrument characteristics, training and leadership.

**Conclusion:**

This historical overview of the development of interRAI instruments in France gives health care organizations pertinent information to guide the implementation of a standardized and comprehensive assessment tool.

## Background

Elderly persons with chronic disorders and greater disability require a combination of health and social long-term care services [1]. This population receives care through a number of institutions such as acute care hospitals, long term care facilities (LTCF) and home care (HC). This fragmentation poses significant challenges to the continuity of care [2]. In many industrialized countries these challenges are amplified by the fragmentation of care systems between several funding agencies [3]. In response to these challenges, an integrated clinical information system using a common language is essential for an efficient transmission of information across all stakeholders [4]. This integrated system should provide standardized and clinically relevant data. It should include a core set of items providing a comprehensive picture of each clients with a direct impact on relevant care plan decisions [5]. A comprehensive needs assessment and care planning across multiple care settings has a positive impact on health parameters and care pathways [6], and has been shown to improve the quality of care [7], resource access [8] and overall performance of the health care system for the elderly population [9].

France has over 66 million inhabitants, with 5.8% of the population older than 80 years and living mainly at home [10]. The French healthcare system is run at the regional level with a single agency governing the delivery of health care, in collaboration with departmental councils at a local level responsible for social services. The French system is still described as fragmented in term of organization, heath production and financing [11] with a decreasing care quality and a lack of continuity of care [12]. Since the end of 1990, the French healthcare system has promoted coordination of care to better address the fragmentation between healthcare and social services [13,14,15] but this effort has been initiated without a standardized comprehensive assessment tool common to all stakeholders. Otherwise, the use of interRAI instruments has increased step by step for twenty years until 2016 and the decision by the Caisse Nationale Solidarité Autonomie (CNSA) to implement the RAI Home Care (HC) instrument for the case managers. The CNSA is the French national agency whose mission is to finance social services, ensure services access and conduct the services organization for people with lost autonomy. In this work we have described the different periods of the interRAI instruments implementation in France, from the onset to mandatory use at the national level. We have identified key incentives and barriers related to this development, providing an overview that will be relevant to other healthcare organizations seeking to implement an integrated information system.

## InterRAI instruments and the potential for integration

The development of the interRAI instruments was part of a set of reforms enacted by the United States Congress in the Omnibus Budget Reconciliation Act of 1987 [16]. Its success made those instruments attractive to the international community of investigators in geriatrics and gerontology topics with more than 35 countries involved as users. The interRAI instruments were developed by the interRAI consortium, a non-profit organization of clinicians, researchers and health administrators from the different countries involved. The goal of the consortium is to improve the quality of life of vulnerable persons through a seamless comprehensive assessment system (www.interrai.org).

The interRAI instruments are a set of standardized and fully structured tools to assess characteristics of people having multiple chronic disorders and receiving long-term care services. Since 1989, the instrument is mandatory in US nursing homes [17] with case mix application [18] to improve the quality of care with the interRAI Long Term Care Facilities (LTCF). The interRAI Home Care (HC) version was created in 1994 and translated in several languages [19]. Other instruments were created for different populations and care settings such as hospital settings (acute care and post-acute care), assisted living, palliative care and mental health for psychiatric patients. In 2001 the interRAI consortium has initiated a reconciliation effort to ensure that all the instruments contained common items and definitions. All instruments now include core items that are shared among instruments, and optional items that are specific to the population or to the care setting. Taken together the interRAI instruments constitute an integrated clinical information system [4].

Each instrument has numerous applications for a wide range of stakeholders such as clinicians, managers and health decision-makers. These applications include Clinical Assessment Protocols (CAPs) for supporting and achieving appropriate care plans, clinical scales for assessing health parameters, quality indicators and case-mix tools [20]. CAPS include triggers to identify persons to whom the protocol applies and a set of guidelines suggesting care planning actions. These actions are driven by the presence of problem conditions, risk factors or potential for improvement. Finally, interRAI instruments are undergoing extensive development and are continuously being tested for validity and reliability by numerous clinicians [21,22,23,24].

## Implementation of the interRAI instruments in France

### First period (1995–2011)

#### Description of the context

At the beginning of the 1990s, the introduction of the interRAI instruments in France resulted from a double move: critics by a group of French scientists working on the concept of functional disability [25], and the need to better respond to the huge fragmentation between health and social care services [12]. At national level the French nursing homes were mandated to improve the quality of care and promote greater functional ability. A Clinical Research Hospital Program (“Programme Hospitalier de Recherche Clinique” PHRC) was conducted between 1996 and 1999, using the interRAI LTCF in eight voluntary settings. The two objectives of this first French research program were to evaluate the acceptability of the instrument by professionals [26], and to review the application of interRAI quality indicators in a routine setting to promote their use [27,28].

In 2000 the non-profit organization interRAI France was set up to promote the interRAI development in France. French back-translation was made for the interRAI-HC and interRAI-LTCF assessment forms, allowing the French team to participate in two European Researches Programs. Between 2001 and 2003, the Ad-HOC study (Aged-Home Care) compared the outcomes of different models of community care services across cities of 11 European countries using interRAI-HC [29]. Between 2009 and 2011, the SHELTER study (Services and Health for Elderly in Long Term care) was designed to implement the InterRAI-LTCF instrument to collect and assess uniform information about nursing home residents across facilities of 7 European countries and Israel. The SHELTER study also confirmed the reliability of the interRAI-LTCF instrument after translation into the languages of the participating countries [30]. Both programs allowed cross national comparisons of home care clients [31], residents [32] and both populations in terms of quality indicators [33,34] and treatment [35]. A service delivery integration index of home care for older persons was proposed, based on process-centered integration and organisational structure approach. Items extracted from the interRAI-HC instrument were used to capture basic characteristics of home care services into a services’ delivery integration index [36].

#### Incentives and barriers

At the national level, these research programs provided a validation of the data collected using the interRAI instruments, and a comparison between clients of different healthcare systems. Improving quality and continuity of care requires a better communication between stakeholders, and constitutes a strong incentive to implement the interRAI instruments as a common language [9]. On the other hand, overlapping responsibilities over clinical practice between stakeholders limited the implementation of the instruments. The target population including clients with unmet needs was an incentive for using the interRAI instrument. Unmet needs were explained by an unrelevant fit between the comprehensive assessment and a non-systematic care planning procedure [37]. This population is characterized by higher resource utilization, and using the RAI instruments could reduce hospitalization from LTCF [7].

Voluntary users of the interRAI instruments were clinicians working in nursing home or community-based care and researchers, with interest in the clinical process of long term care including comprehensive assessment and care planning. Other users such as managers and decisions makers were not involved in the development of the interRAI instruments during this first period. According to these initial providers, interRAI instruments were perceived as promoting better assessment and care plan and better care knowledge with the participation of the resident and caregiver in multidisciplinary care team [26]. The implementation of interRAI instruments also helped identifying a number of issues in the organization of work. When combined with the long-term use of home-made tools and the fear of practice standardization, some resistance grew over the fear of losing one’s professional autonomy [38]. There was also a lack of connections between research purpose and clinical utilization without incentives to promote a routinely use.

While the quality of the data captured and the direct clinical applicability were incentives, the time required for the complete clinical assessment, based on 280 items, proved too long for some providers. This resistance was partially addressed through a 4-day training explaining the functional approach of the evaluation, but the use of paper forms or the need for access to a computer to use the CAPS and Scales algorithms remained key limitations. The creation of the InterRAI France was an important factor to structure the development of the interRAI instruments. The leadership was represented by a public health physician (JCH) who participates at interRAI consortium into the System Development Committee. It allowed the creation of French versions of the instruments with corresponding manuals. The absence of an academic clinician physician in the leadership team leading to a limited engagement of clinicians from other settings precluded the development of the instruments (Table 1).

**Table 1:** Incentives and barriers of the implementation of the RAI Instruments first period.

	Period 1	
	Incentives	Barriers

**National context**	Comparative research programs	Overlapping clinical practices between stakeholders
	Better quality of care and information provided	
**Target Population**	LTCF residents and older community care clients with unmet needs	
**Persons involved**	Voluntary clinicians and researchers	Managers and decisions makers not involved
**Impacts on providers**	Better assessment of residents needs	Existence of internal assessment instrument
	Participation of residents and caregivers	Fear of practice standardization
	Reinforcement of the multidisciplinary care team	Issues with work organization
	Improvement of gerontology knowledge	Lack of connection between research and the clinics
**RAI characteristics**	Quality of data	Time required for assessment using all 280 items
	Unique assessment with series of applications	
**Training**	4-days training	No electronic version of the RAI during clinical studies
**Leadership**	InterRAI France organization	No clinical physicians involved in leadership
	Participation of an academic public health physician in InterRAI consortium	

### Second period (2009–2015)

#### Description of the context

In 2009, the French government implemented an integrated care model for elderly population at the national level [39]. Integrated care is a collective approach involving all stakeholders in a defined territory, addressing the fragmentation of services through interdependent mechanisms and tools [40,41]. These included 6 components such as areas for cooperation, a shared access-to-services process, case management for older persons with complex needs, a common instrument for comprehensive assessment, planning of care services and information sharing system [42,43]. The implementation of this integrated care organization was set up in 17 experimental sites between 2009 and 2012. Each site had to choose a comprehensive assessment tool for their case managers among the following three: GEVA-A (Guide d’EVAluation des besoins des personnes Agées [Guide to EVAluation of the needs of Aged individuals]), developed by French authorities; l’OEMD-SMAF (Outil d’Evaluation MultiDimensionnel basé sur le Système de Mesure de l’Autonomie Fonctionnelle [multidimensional evaluation tool based on the system for measuring functional autonomy]), a French adaptation of the Quebec tool; and the interRAI-HC. The city of Marseille was the first experimental site to use the interRAI-HC on a large scale.

In 2011, the Pertinence des Outils d’Evaluation Multidimensionnelle (POEM) study assessed the use of the 3 tools by case managers. Among the recommendations made were the need for a standardized and multidimensional assessment tool, available as an electronic version with an appropriate 2-phase training [44]. Between 2012 and 2017 the integrated care model has been extended to 352 sites covering 98% of the national territory. Each site was chosen based on inhabitants and healthcare and service organizations.

#### Incentives and barriers

At the national level, the integrated care program run at a national pilot was a strong incentive [40]. The step-by-step implementation combined with an experimental phase gave some indications as to how and where the integrated care model had to be provided [41]. However the level of integration, defined as the level of implementation of the 6 components of the integrated model of care, remained low. The target population of this program was mainly the elderly population characterized by complex interactions between social, physical, psychological, economic, and environmental dimensions.

In the context of the integrated care model case managers were appointed to address the specific needs of this population using a 5-step process: identifying people with complex needs, standardized multidimensional assessment of needs, establishment of a care plan and follow-up with regular needs reassessment and care plan adaptation [45]. This case management approach provides an operating model to implement a multidimensional tool such as the RAI-HC. It allows collects information on the population needs and supports the care planning through CAPS guidelines. During the experimental phase, only the case managers had to use a standardized comprehensive assessment tool. While this helped them form their new professional identity and reinforced their legitimacy, it also introduced a difference with other care providers who were not requitred to use them [44]. Moreover, case managers did not use other applications such as quality indicators and RUGs, thus limiting the benefits related to the full deployment of the instruments.

The standardization allowed case managers to homogenize practices and avoided confusion in the interpretation of items (same definitions and coding). The 5-days training associated with a continuous support in the experimental territory reinforced the appropriation of the tool by case managers. The electronic version was available and allowed assessors to get a direct access to scales and CAPS. The participation of geriatricians in InterRAI France reinforced the leadership of the organization (Table 2).

**Table 2:** Incentives and barriers of the implementation of the RAI Instruments second period.

	Phase 2	
	Incentives	Barriers

**National context**	Integrated model of care with a national pilot	Low integration level
	Step by step implementation	
**Target Population**	Older people with complex health and social needs	
**Persons involved**	Voluntary community-based case managers	No other users
**Impacts on providers**	Improving the case management process with the care planning	Other stakeholders were using different tools
	Legitimation of the role of CM	Lack of appropriation of other applications of the instruments
**RAI instrument characteristics**	Standardized and homogenized data collection	
**Training**	5 days training and local support	
	Electronic version of the instruments	
**Leader ship**	Mixed leadership of academic and clinical physicians	

### Third part: the call for tenders

In 2015, a national call for tenders was issued to choose a standardized comprehensive assessment tool for case managers.

The criteria for tool choice were:

– existence of a conceptual framework with an international functional classification
– a multidimensionality approach
– relevance to older people with loss of autonomy
– possibility to perform the assessment at home
– relevance to care planning
– overview of resource utilization
– scientific validity
– existence of an international network
– active development.

In 2016, interRAI-HC was choosen by the CNSA as the mandatory instrument for the 1000 case managers working in the integrated care model implemented in the entire territory.

## Discussion

The research-driven approach for improving quality of care, the move from coordination to an integrated care model and the step by step implementation of case management were the main national factors that contributed to the success of interRAI instruments together with their positive impact on providers, their scientific validity and relevance, and the interdisciplinary team driving their development.

At the international level, studies conducted by the interRAI consortium have driven the improvement of the quality of data produced and developed the range of instruments. At the French level, while clinicians were strongly involved in clinical studies, the tools were not implemented in routine clinical. To drive adoption, managers and decisions makers had to be involved, and potential barriers from the providers at local level had to be anticipated. Despite initial resistance, the interRAI instruments are now used in the clinics, and we are foreseeing a strong development of a research based on the data collected by 1000 case managers, each with a case load of 40 persons and a minimum of 2 assessments a year. This dataset provides an unprecedented view into the elderly population that has the potential to drive key changes in the standard of care. Collecting and anonymizing this data and transferring it to interRAI France and interRAI consortium are key challenges to the sustainability of this international effort. Future efforts will need to include incentives not only for the clinical users of the instruments, but also for software developers who are implementing the electronic record systems supporting data collection.

The development of the integrated care program in France combined a bottom-up and a top-down approach. It means a double piloting with the presence of a pilot at the national level and at the local level. The national pilot required an active participation of the local sites, and was driven by a better comprehension of the needs of all stakeholders from the healthcare and social services [46]. The implementation of interRAI-HC within an integrated care model will require adoption of the instruments by all local stakeholders. We suggest the implementation of screener instruments as a way to promote the adoption of other interRAI instruments and attract new providers. For example, the interRAI Contact Assessment (CA), a screener based on common collapsed items of interRAI-HC, identifies which potential home care clients should be referred for a full assessment or for services such as rehabilitation [47]. The interRAI-CA is relevant for providers in LTCF, palliative care, acute and post-acute care hospital and skilled nursing home facility. Ultimately, the availability of a common standardized comprehensive tool allows for the construction of an integrated clinical information system and will drive the integration of care between all services.

The creation of the non-profit organization interRAI France was instrumental to the development of interRAI instruments in clinical practice in France. InterRAI France is involved in the construction of training programs for the different providers, runs the network of users and supports them in the appropriation of the tools. Several members of InterRAI France participate at the interRAI consortium. They are responsible for the dissemination of relevant information from local providers to the international network. This drives the constant improvement of items and instruments that is key to the success to the platform. interRAI France is driven by a mixed team of clinicians and non-clinicians, who provide a strong support to promote the instruments and engage with a larger number of stakeholders. It has been shown that this double leadership is a strong incentive for supporting the case management, reinforcing the inter-professional bonds and changing professional practices [48].

Our review of the results of different studies in the context of the development of an integrated care model is relevant to healthcare professionals and organizations of other countries planning a similar transition. The incentives, as well as the solutions that were identified to overcome stakeholder resistance, are important guidelines to the development and improvement of a standardized and complete integrated clinical information system.

## References

[1] Min, LC, Elliott, MN, Wenger, NS and Saliba, D. Higher vulnerable elders survey scores predict death and functional decline in vulnerable older people. Journal of American Geriatrics Society, 2006; 54(3): 507–511. DOI: 10.1111/j.1532-5415.2005.00615.x 16551321

[2] Kahn, JM and Angus, DC. Going home on the right medications: Prescription errors and transitions of care. Journal of the American Medical Association, 2011; 306: 878–879. DOI: 10.1001/jama.2011.1209 21862750

[3] Leichsenring, K. Developing integrated health and social care services for older persons in Europe. International Journal of Integrated Care, 2004; 4: e10 Epub 2004 Sep 3. DOI: 10.5334/ijic.107 16773149 PMC1393267

[4] Gray, LC, Berg, K, Fries, BE, Henrard, JC, Hirdes, JP and Steel, K. Sharing clinical information across care settings: the birth of an integrated assessment system. BMC Health Services Research, 2009; 9: 71 DOI: 10.1186/1472-6963-9-71 19402891 PMC2685125

[5] Beswick, AD, Rees, K, Dieppe, P, Ayis, S, Gooberman-Hill, R, Horwood, J and Ebrahim, S. Complex interventions to improve physical function and maintain independent living in elderly people: a systematic review and meta-analysis. Lancet, 2008; 371: 725–35. DOI: 10.1016/S0140-6736(08)60342-6 18313501 PMC2262920

[6] Mor, V, Intrator, O, Fries, BE, Phillips, C, Teno, J, Hiris, J, Hawes, C and Morris, J. Changes in hospitalization associated with introducing the Resident Assessment Instrument. Journal of American Geriatrics Society, 1997; 45(8): 1002–1010. DOI: 10.1111/j.1532-5415.1997.tb02973.x 9256855

[7] Mor, V. Improving the quality of long-term care with better information. Milbank Quarterly, 2005; 83(3): 333–36. DOI: 10.1111/j.1468-0009.2005.00405.x 16201996 PMC2690144

[8] Fries, BE, Fahey, CJ, Hawes, C, Vladeck, BC, Morris, J, Phillips, C, Fredeking, H, Hirdes, J, Sinclair, DG and King, JT. Implementing the Resident Assessment Instrument: Case studies of policymaking for long-term care in eight countries. Milbank Memorial Fund, 2003 10; 129 https://www.milbank.org/publications/implementing-the-resident-assessment-instrument-case-studies-of-policymaking-for-long-term-care-in-eight-countries/.

[9] Hofmarcher, MM, Oxley, H and Rusticelli, E. Improved health system performance trough better care coordination. OECD Health working papers, 2007; 30 https://www.oecd.org/els/health-systems/39791610.pdf.

[10] Dos Santos, S and Makdessi, Y. Une approche de l’autonomie chez les adultes et les personnes âgées [evaluation of functional autonomy in adult and aging population]. Etudes et Résultats DRESS; 2010 http://drees.solidarites-sante.gouv.fr/IMG/pdf/er718.pdf [in French].

[11] Henrard, JC, Ankri, J and Le Disert, D. Soins et aides aux personnes âgées. Effets des caractéristiques structurelles du système sur la mise en oeuvre de la politique et le fonctionnement des services [Relations between political approach and the organisation of healthcare services]. Sciences Sociales et Santé, 1991; 9(1): 39–54. [in French]. DOI: 10.3406/sosan.1991.1183

[12] HCAAM. Assurance maladie et perte d’autonomie. Contribution au débat sur la dépendance des personnes âgées [health insurance and autonomy loss for elderly population]. Rapport du 23 juin, 2011 [in French]. http://www.securitesociale.fr/IMG/pdf/hcaam_rapport_assurance_maladie_perte_autonomie.pdf.

[13] Frossard, M, Genin, N, Guisset, M and Villez, A. Providing integrated health and social care for older persons in France – an old idea with a great future In: Providing integrated health and social services for older persons, Billings, J and Leichsenring, K (eds.), 2004; 229–68. Aldershot, UK: Ashgate.

[14] Somme, D and de Stampa, M. Ten years of integrated care for the older in France. International Journal Integrated Care [serial online], 2011 1; 11 (Spec 10th Anniversary Ed): e141. Epub 2011 Dec 14, 2011 Dec 14; 11. 10.5334/ijic.668 PMC3284287 22375101

[15] Henrard, JC. Le système français d’aide et de soins aux personnes âgées [the health and social care system for older persons in France]. Santé Société et Solidarité, 2002; 2: 73–82. [in French]. DOI: 10.3406/oss.2002.894

[16] Hawes, C, Mor, V, Phillips, CD, Fries, BE, Morris, JN, Steele-Friedlob, E, Greene, AM and Nennstiel, M. The OBRA-87 nursing home regulations and implementation of the Resident Assessment Instrument: Effects on process quality. Journal of American Geriatrics Society, 1997 8; 45(8): 977–85. DOI: 10.1111/j.1532-5415.1997.tb02970.x 9256852

[17] Hawes, C, Morris, JN, Phillips, CD, Fries, BE, Murphy, K and Mor, V. Development of the nursing home Resident Assessment Instrument in the USA. Age and Ageing, 1997; 26(S2): 19–25. 9464550 10.1093/ageing/26.suppl_2.19

[18] Fries, BE, Schneider, DP, Foley, WJ, Gavazzi, M, Burke, R and Cornelius, E. Refining a case-mix measure for nursing homes: Resource Utilization Groups (RUG-III). Medical Care, 1994; 32(7): 668–685. DOI: 10.1097/00005650-199407000-00002 8028403

[19] Morris, JN, Fries, BE, Steel, K, Ikegami, N, Bernabei, R, Carpenter, GI, Gilgen, R, Hirdes, JP and Topinková, E. Comprehensive clinical assessment in community setting: Applicability of the MDS-HC. Journal of American Geriatrics Society, 1997; 45: 1017–24. DOI: 10.1111/j.1532-5415.1997.tb02975.x 9256857

[20] Berg, K, Finne-Soveri, H, Gray, L, Henrard, JC, Hirdes, J, Ikegami, N, Ljunggren, G, Morris, JN, Paquay, L, Resnik, L and Teare, G. Relationship between interRAI HC and the ICF: Opportunity for operationalizing the ICF. BMC Health Services Research, 2009; 9: 49 DOI: 10.1186/1472-6963-9-47 19292897 PMC2666676

[21] Morris, J, Hawes, C, Fries, B, Philips, C, Mor, V, Katz, S, Murphy, K, Drugovich, ML and Friedlob, AS. Designing the National Resident Assessment Instrument for nursing Homes. Gerontologist, 1990; 30: 293–302. DOI: 10.1093/geront/30.3.293 2354790

[22] Bernabei, R, Landi, F, Gambassi, G, Sgadari, A, Zuccala, G, Mor, V, Rubenstein, LZ and Carbonin, P. Randomised trial of impact of model of integrated care and case management for older people living in the community. British Medical Journal, 1998; 316: 1348–1351. DOI: 10.1136/bmj.316.7141.1348 9563983 PMC28532

[23] Landi, F, Tua, E, Onder, G, Carrara, B, Sgadari, A, Rinaldi, C, Gambassi, G, Lattanzio, F and Bernabei, R. SILVERNET-HC Study Group of Bergamo. Minimum Data Set for home care: A valid instrument to assess frail older people living in the community. Medical Care, 2000; 38: 1184–1190. DOI: 10.1097/00005650-200012000-00005 11186297

[24] Ribbe, M, Ljunggren, G, Steel, K, Topinkova, E, Hawes, C, Igegami, N, Henrard, JC and Jonnson, P. Nursing homes in 10 nations: A comparison between countries and settings. Age Ageing, 1997; 26, 3–12. DOI: 10.1093/ageing/26.suppl_2.3 9464548

[25] Gardent, H. A propos de l’évaluation de la dépendance [assessment of the dependency]. Gérontologie et Société, 1993; 65: 16–23. [in French].

[26] Garreau, H. Etude de la perception des utilisateurs d’une nouvelle démarche d’évaluation du résidant, le Resident Assessment Instrument, experimenté dans des établissements de soins de longue durée [perceptions of RAI utilisation in long term care facilities] Mémoire de DESS, 2001; Laboratoire Universitaire Santé et Vieillissement (EA 2506) [in French].

[27] Henrard, JC, Cérase-Feurra, V and Ankri, J. Intérêt du Resident Assessment Instrument (RAI) pour l’évaluation de la qualité des soins de longue durée [interest of using the RAI approach for quality of care in long term care facilities]. La Revue de Gériatrie, 2000; 25(4): 231–242. [in french].

[28] Moty, C, Barberger-Gateau, P, De Sarasqueta, AM, Teare, GF and Henrard, JC. Ajustement d’indicateurs de qualité des soins pour les Etablissements Hébergeant des Personnes Agées Dépendantes en France: une étude préliminaire [Risk adjustment of quality indicators in French long term care facilities for elderly people. A preliminary study]. Revue d’Epidémiologie et de Sante Publique, 2003; 51(3): 327–338. [in French]. 13130213

[29] Carpenter, I, Gambassi, G, Topinkova, E, Schroll, M, Finne-Soveri, H, Henrard, JC, Garms-Homolova, V, Jonsson, P, Frijters, D, Ljunggren, G, Sørbye, LW, Wagner, C, Onder, G, Pedone, C and Bernabei, R. Community care in Europe. The Aged in Home Care project (AdHOC). Aging Clinical Experimental Research, 2004 8; 16(4): 259–69. DOI: 10.1007/BF03324550 15575119

[30] Onder, G, Carpenter, GI, Finne-Soveri, H, Gindin, J, Frijters, D, Henrard, JC, Nikolaus, T, Topinkova, E, Tosato, M, Liperoti, R, Landi, F and Bernabei, R. SHELTER project. Assessment of nursing home residents in Europe: the Services and Health for Elderly in Long TERm care (SHELTER) study. BMC Health Services Research, 2012; 12(1): 5 DOI: 10.1186/1472-6963-12-5 22230771 PMC3286368

[31] Sørbye, LW, Garms-Homolová, V, Henrard, JC, Jónsson, PV, Fialova, D, Topinkova, E and Gambassi, G. Shaping home care in Europe: The contribution of the Aged in Home Care project. Maturitas, 2009; 62(3): 235–42. DOI: 10.1016/j.maturitas.2008.12.016 19181465

[32] Yamada, Y, Denkinger, MD, Onder, G, van der Roest, HG, Finne-Soveri, H, Bernabei, R and Topinkova, E. Joint Associations of Dual Sensory Impairment and No-Activity Involvement With 1-Year Mortality in Nursing Homes: Results from the SHELTER Study. The Journals of Gerontoly Serie A Biological Sciences and Medical Sciences, 2016 5; 71(5): 643–8. DOI: 10.1093/gerona/glv191 26582074

[33] Bos, JT, Frijters, DHM, Wagner, C, Carpenter, GI, Finne-Soveri, H, Topinkova, E, Garms-Homolová, V, Henrard, JC, Jónsson, PV, Sørbye, L, Ljunggren, G, Schroll, M, Gambassi, G and Bernabei, R. Variations in quality of Home Care between sites across Europe as measured by Home Care Quality Indicators. Aging Clinical Experimental Research, 2007; 19(4): 323–329. DOI: 10.1007/BF03324709 17726364

[34] Frijters, DH, Van der Roest, HG, Carpenter, IG, Finne-Soveri, H, Henrard, JC, Chetrit, A, Gindin, J and Bernabei, R. The calculation of quality indicators for long term care facilities in 8 countries (SHELTER project). BMC Health Services Research, 2013; 13: 138 DOI: 10.1186/1472-6963-13-138 23587337 PMC3716904

[35] Foebel, AD, Liperoti, R, Gambassi, G, Gindin, J, Ben Israel, J, Bernabei, R and Onder, G. Prevalence and correlates of cardiovascular medication use among nursing home residents with ischemic heart disease: Results from the SHELTER study. Journal of American Medicine Direction Association, 2014; 6; 15(6): 410–5. DOI: 10.1016/j.jamda.2013.12.085 24559641

[36] Henrard, JC, Ankri, J, Frijters, D, Carpenter, I, Topinkova, E, Garms-Homolova, V, Finne-Soveri, H, Sørbye, LW, Jónsson, PV, Ljunggren, G, Schroll, M, Wagner, C and Bernabei, R. Proposal of a service delivery integration index of home care for older persons: application in several European cities. International Journal of Integrated Care, 2006; 6: e11 DOI: 10.5334/ijic.159 17006549 PMC1570876

[37] Asch, SM, Sloss, EM, Hogan, C, Brook, RH and Kravitz, RL. Measuring underuse of necessary care among elderly Medicare beneficiaries using inpatient and outpatient claims. Journal of the American Medical Association, 2000; 284: 2325–2333. DOI: 10.1001/jama.284.18.2325 11066182

[38] Couturier, Y, Trouve, H, Gagnon, D, Etheridge, F, Carrier, S and Somme, D. Receptivité d’un modèle québécois d’intégration des services aux personnes âgées en perte d’autonomie en France [Recetivity of a quebecois integrated care model in France]. Lien Social et Politiques, 2000; 62: 163–174 [in French].

[39] Présidence de la République. Plan Alzheimer et maladies apparentées 2008–2012. [Alzheimer’s disease and related disorders” 2008–2012 plan]. 2008 2 1 Available from: www.sante.gouv.fr/IMG/pdf/Plan_Alzheimer_ 2008-2012-2.pdf [in French].

[40] de Stampa, M and Somme, D. Rapport d’expertise 2010–2011 sur la phase expérimentale des MAIA – Plan National Alzheimer.[Expertise report on 2010–2011 phase of the MAIA experimentation – National Alzheimer Plan.]. 2012 2 1 [cited 2013 Jul 4]. www.ladocumentationfrancaise.fr/var/storage/rapports-publics/124000109/0000.pdf [in French].

[41] de Stampa, M and Somme, D. Rapport d’expertise 2011–2012 sur la phase expérimentale des MAIA – Plan National Alzheimer. [Expertise report on 2011–2012 phase of the MAIA experimentation – National Alzheimer Plan.]. 2012 2 1 [cited 2013 Jul 4]. www.ladocumentationfrancaise.fr/var/storage/rapports-publics/124000109/0000.pdf [in French].

[42] Hebert, R, Durand, PJ, Dubuc, N and Tourigny, A. PRISMA: A new model of integrated service delivery for the frail older people in Canada. International Journal of Integrated Care, 2003; 3: e08 www.ijic.org/index.php/ijic/article/view/73/146 DOI: 10.5334/ijic.73 16896376 PMC1483944

[43] Béland, F, Bergman, H, Lebel, P, Dallaire, L, Fletcher, J, Contandriopoulos, AP and Tousignant, P. Integrated services for frail elders (SIPA): a trial of a model for Canada. Canadian Journal of Aging, 2006; 25(1): 5–42. Spring DOI: 10.1353/cja.2006.0018 16770746

[44] Nugue, M, de Stampa, M, Couturier, Y and Somme, D. Tools and the Emergence of the Case Manager’s Professional Identity in France. Care Management Journal, 2012; 13(4): 184–93. DOI: 10.1891/1521-0987.13.4.184 23383583

[45] Challis, D, Darton, R, Hughes, J, Stewart, K and Weiner, K. Intensive care-management at home: an alternative to institutional care? Age Ageing, 2001; 30(5): 409–413. DOI: 10.1093/ageing/30.5.409 11709380

[46] Vedel, I, de Stampa, M, Bergman, H, Ankri, J, Cassou, B, Blanchard, F and Lapointe, L. Healthcare professionals and managers’ participation in developing an intervention: A pre-intervention study in the elderly care context. Implement Science, 2009; 4(1): 21 DOI: 10.1186/1748-5908-4-21 PMC2678079 19383132

[47] Cheng, L, Zhu, M, Poss, JW, Hirdes, JP, Glenny, C and Stolee, P. Opinion versus practice regarding the use of rehabilitation services in home care: an investigation using machine learning algorithms. BMC Medicine Information Decision, 2015 10 9; 15: 80 DOI: 10.1186/s12911-015-0203-1 PMC4600209 26453354

[48] de Stampa, M, Vedel, I, Mauriat, C, Bagaragaza, E, Routelous, C, Bergman, H, Lapointe, L, Cassou, B, Ankri, J and Henrard, JC. Diagnostic study, design and implementation of an integrated model of care in France: a bottom-up process with continuous leadership. International Journal of Integrated Care, 2010; 10: e034 DOI: 10.5334/ijic.506 20216954 PMC2834925

